# Data for proteomic analysis of Human monocyte-derived macrophages

**DOI:** 10.1016/j.dib.2015.05.012

**Published:** 2015-05-26

**Authors:** S. Eligini, M. Brioschi, S. Fiorelli, E. Tremoli, S. Colli, C. Banfi

**Affiliations:** **a** Centro Cardiologico Monzino I.R.C.C.S, Milan, Italy; **b** Dipartimento di Scienze Farmacologiche e Biomolecolari, Università degli Studi di Milano, Milan, Italy

**Keywords:** Macrophages, Proteomics, Laser capture microdissection

## Abstract

This data article is referred to the research article entitled *Human monocyte-derived macrophages are heterogeneous: proteomic profile of different phenotypes* by Eligini et al. Eligini S., Brioschi M., Fiorelli S., Tremoli E., Banfi C., Colli S. Human monocyte-derived macrophages are heterogeneous: proteomic profile of different phenotypes. J. Proteomics 124, 2015, 112-123. Macrophages obtained in vitro from blood monocytes are largely used as surrogate model of tissue macrophages that are heterogeneous and not easy to obtain and handle. Under spontaneous differentiation in vitro*,* monocyte-derived macrophages (MDMs) display two dominant subsets (round and spindle) that show different transcriptional, antigenic, and functional profiles mimicking, at least in part, the heterogeneity of tissue macrophages. This article reports the nano-LC-MS^E^ analysis of the proteome of round and spindle MDMs allowing a deeper comprehension of macrophage heterogeneity.

**Table:** Specifications Table

Subject area	Biology
More specific subject area	Cellular proteomics
Type of data	Excel files
How data was acquired	Experiments were performed by means of the hybrid quadrupole-time of flight mass spectrometer SYNAPT-G1 (Waters Corporation, Milford, MA, USA) coupled to the nanoAQUITY UPLC system (Waters Corporation, Milford, MA, USA).
Data format	Processed data
Experimental factors	Human monocytes were spontaneously differentiated towards macrophages for 7 days in the presence of autologous serum. Two dominant and distinct morphotypes (round and spindle) were found to co-exist in the same culture plate.
Experimental features	Round or spindle MDMs were singly dissected by laser capture microdissection, digested with trypsin, analysed by nano-LC-MS^E^ and processed with PLGS 2.3 (Waters Corporation, Milford, MA, USA.)
Data source location	Milan, Italy
Data accessibility	*Data are provided with this article and referred to*[1]

**Value of the data**

• Mass spectrometry based analysis of human MDM morphotypes was performed with a minimal number of cells (6000 MDM/morphotypes) singly isolated by laser capture microdissection
• A total of 132 proteins were identified within the MDM proteomes using a label free MS-based proteomic approach
• Here we show data about the proteome that characterises the two dominant phenotypes of human MDMs generated by spontaneous differentiation from blood-derived monocytes

Data, experimental design, materials and methods

Human monocytes were isolated from healthy subjects and differentiated in vitro towards macrophages. Two dominant and distinct morphotypes (round and spindle) were found to co-exist in the same culture plate in an approximately ratio of 1:1. Round or spindle MDMs were singly isolated from cell culture dishes by laser capture microdissection (LCM) in order to analyse the distinct proteomic profiles. After trypsin digestion, their proteomes were analysed by a label free nano-LC-MS^E^ analysis which allowed a qualitative and quantitative analysis of the proteins (Table S1) identified from 2261 and 2428 peptides (Table S2) in round and spindle MDMs, respectively.

## 1 Materials and methods

### 1.1 Cell cultures

Human peripheral blood mononuclear cells were isolated from venous blood of healthy consenting volunteers, as described in [2], and cultured over 7 days in Medium 199 (Lonza, EuroClone, Milan, Italy) supplemented with 10% autologous serum.

### 1.2 Laser capture microdissection (LCM)

Air-fixed MDMs were singly microdissected with the laser microdissection system from PALM MicroLaser Technologies (Bernried, Germany), harvesting about 6000 cells for each morphotype in order to perform the proteomic analysis as previously described [3]. Briefly, after capture by the RoboPC׳s autocatapulting feature (UV-Energy 90–100 and UV-Focus 42), macrophages were catapulted directly into the cap of a 0.65 mL microcentrifuge tube. Approximately 6000 laser-pulsed cells of both morphotypes were isolated from a total of 14 healthy subjects, limiting the use of the same culture for no more than 3 days. LCM isolated cells were then stored at −80 °C till processing.

### 1.3 Label-free LC-MS^E^ analysis

LCM isolated cells present in the tube׳s cap were dissolved in 25 mmol/L NH_4_HCO_3_ containing 0.1% RapiGest (Waters Corporation, Milford, MA, USA) and recovered in the tube with a short spin. Pools of cells with the same morphology were then sonicated and centrifuged at 13,000×*g* for 10 min. After heating at 80 °C for 15 min, each sample was reduced with 5 mmol/L dithiothreitol (DTT) at 60 °C for 15 min, and then carbamidomethylated with 10 mmol/L iodacetamide for 30 min at room temperature. Digestion was performed over-night at 37 °C, maintaining a ratio of 1 µg of trypsin (Promega, Milan, Italy)/20 µg of protein, based on the calculated recovery for the number of processed cells [3]. Trifluoroacetic acid (2% v/v) was added after digestion to hydrolyse the RapiGest and inactivate the trypsin. Peptide concentration and purification were performed using ZipTip C18 (Millipore, Milan, Italy) in accordance with the manufacturer׳s instructions and the peptides were then solubilised in 0.1% v/v formic acid in water.

The tryptic peptides obtained from protein digestion of LCM isolated MDMs were analysed by LC-MS^E^ by means of a nanoACQUITY system coupled to a SYNAPT-MS, a hybrid Q-TOF mass spectrometer (Waters Corporation, Milford, MA, USA), as previously described [3]. ProteinLynx GlobalSERVER (PLGS) v 2.3 (Waters Corporation, Milford, MA, USA) was used for ion detection, data clustering, and database search of the data-independent LC-MS^E^ data, as previously explained in detail [4–6]. The entire data set of identified proteins was filtered by considering only those with more than 2 identified peptides, replicating in at least two out of three technical instrument replicates.

## Footnotes

Appendix A Supplementary data associated with this article can be found in the online version at doi:10.1016/j.dib.2015.05.012.
